# A spiral plasmonic lens with directional excitation of surface plasmons

**DOI:** 10.1038/srep32345

**Published:** 2016-08-26

**Authors:** Qingrui Guo, Chi Zhang, Xinhua Hu

**Affiliations:** 1 Department of Materials Science, Laboratory of Advanced Materials and Key Laboratory of Micro and Nano Photonic Structures (Ministry of Education), Fudan University, Shanghai 200433, China

## Abstract

Conventional plasmonic lenses are composed of curved slits carved through metallic films. Here, we propose a new plasmonic lens based on a metallic slit with an auxiliary groove. When the lens is illumined normally, only inward surface plasmon polaritons (SPPs) can be generated and then focused into a hot spot at the center of the lens. The focusing effect is theoretically investigated by varying the groove parameters and incident polarizations. It is found that this phenomenon exists for both the circular and linear polarizations of incidence. Under optimal groove parameters, the intensity of the focal spot in our lens can be 2.5 times of that in one without grooves for both linearly and circularly polarized illuminations.

Surface plasmon polaritons (SPPs), surface electromagnetic waves propagating at the interface between dielectric and metallic layers^1^, have received much attention recently due to their promising applications such as plasmon focusing^2^,^3^,^4^,^5^,^6^,^7^,^8^,^9^,^10^,^11^,^12^, subwavelength waveguiding^13^,^14^,^15^, near-field imaging and sensing^1^,^16^.

SPPs can be generated at a metallic film that has a slit and is illuminated by a light beam from vacuum^17^,^18^,^19^. When the slit is curled into a round shape, SPPs can be further focused into the central area of the curled slit^2^. The size of the focal spot can be much smaller than the incident wavelength when a spiral slit or a pair of half-circle slits are applied^3^,^4^,^5^,^10^,^11^,^12^. Such metallic structures with curled slits can serve as plasmonic lenses, holding applications in maskless lithography^9^ and microscopy^11^.

Current plasmonic lenses convert optical waves in vacuum into both inward and outward SPPs, of which the latter could reduce the efficiency of focusing. When a lens array is introduced, outward SPPs can interfere in the areas outside lenses. Such drawbacks can be solved by replacing metallic slits with chains of rotated nanoapertures^20^,^21^. However, this approach works only for incident light with a certain circular polarization and is invalid for linear and another circular polarizations. It would be interesting to know whether there exists a scheme that is valid for all the circular and linear polarizations.

In this paper, we show that, via using a metallic slit with an auxiliary groove in plasmonic lenses, outward SPPs can be eliminated for all the circular and linear polarizations of incidence. It is found that the parameters of grooves can influence dramatically on the focusing effect of lenses. By using a global search, optimal groove parameters are obtained to achieve superior focusing performance.

## Results

### 2D structures with and without directional excitation of SPPs

**Figure 1:**
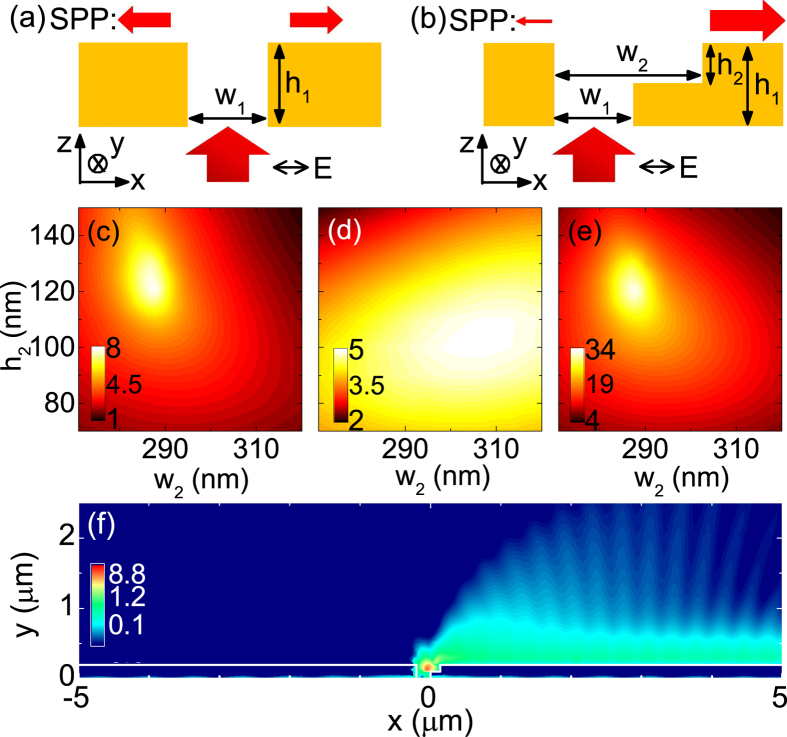
Directional excitation of SPPs by an asymmetric plasmonic nanoslit. (**a**) Side view of a plat Au film with a slit that has a width of *w*_1_ = 150 nm and is along the *y* direction. The thickness of the film is *h*_1_ = 250 nm. (**b**) The same as (**a**) but for a structure with an auxiliary groove with a width of *w*_2_ − *w*_1_ and depth of *h*_2_. When the slits are illuminated from the lower side by a 2D Gaussian light beam with *x*-polarization, wavelength *λ*, width of *w*_*g*_ = 7 *μ*m, and |**E**| = *p* in center, leftward and rightward SPPs can be excited in the upper side with the same [different] intensities in (**a**) [(**b**)]. (**c–e**) ln(*E*_*R*_), *I*_right_, and excitation factor *F*_*E*_ = *I*_right_ln(*E*_*R*_) (shown in color) as a function of *w*_2_ and *h*_2_, respectively. Here, *λ* = 700 nm and extinction ratio *E*_*R*_ = *I*_right_/*I*_left_, where *I*_right_ and *I*_left_ are the rightward and leftward SPP intensities in (**b**), respectively. The excitation factor *F*_*E*_ reaches a maximum at optimal parameters of *w*_2_ = 288 nm and *h*_2_ = 120 nm. (**f**) Logarithmic simulated profile of |**E**_*z*_|^2^/*p*^2^ for the optimal structure obtained in (**e**).

Figure 1(a) shows a conventional plasmonic antenna, which is a slit on a plat Au film and along the *y* direction. When the slit is illuminated by a 2D Gaussian light beam with *x*-polarization from the bottom, SPPs can be excited on the upper surface of the Au film. For such a symmetric slit, equal intensities of SPPs can be launched to both the left and right directions. In the followings, we consider Au films with thickness of *h*_1_ = 250 nm, slits with width of *w*_1_ = 150 nm, and a Gaussian light beam with wavelength *λ* = 700 nm, width of *w*_*g*_ = 7 *μ*m, and |**E**| = *p* in center. We note that SPPs can also be excited at the upper surface of the Au film when the slit is replaced by a groove and impinged by a light from the top^22^,^23^. But in this scheme, both the incident and reflected light beams exist above the Au film, which may influence the performance of the corresponding plasmonic lens.

In order to generate unidirectional SPPs, the metallic slit in Fig. 1(a) is replaced by one with an auxiliary metallic groove, which has a width of *w*_2_ − *w*_1_ and depth of *h*_2_ [Fig. 1(b)]. For such an asymmetric structure, its upper part acts as a Fabry-Perot nanocavity, and leftward and rightward SPPs can possess different intensities^24^. Although our structure is similar to the one in ref. 22, its underlying physical mechanism is different from that in ref. 22 [see ref. 24]. We apply a finite-element method to simulate the structure, where the dielectric constant of gold *ε*_Au_ is from experimental data^25^. For incident wavelength *λ* = 700 nm, *ε*_Au_ = −15.2 + 1.24*i*, resulting in wavelength *λ*_SPP_ = *λ*(*ε*_Au_ + 1)/*ε*_Au_]^1/2^ = 677 nm for SPPs at the air-Au interface.

The intensities of leftward and rightward SPPs are defined as 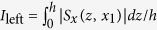 and 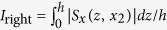, respectively. Here, *S*_*x*_ is the time-average power flow along the *x* direction, *h* = 0.3 *μ*m, *z* = 0 is at the upper surface of the Au film, *g* = 2 *μ*m, and *x* = *x*_1_ + *g* and *x* = *x*_2_ − *g* are at the left and right boundaries of the slit, respectively. An extinction ratio *E*_*R*_ and excitation factor *F*_*E*_ are also defined as:


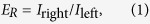



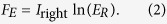


We note that a large excitation factor *F*_*E*_ relates to both a large *I*_right_ and small *I*_left_.

In Fig. 1(c–e), ln(*E*_*R*_), *I*_right_, and *F*_*E*_ are plotted as a function of *w*_2_ and *h*_2_, respectively. We can see that excitation factor *F*_*E*_ reaches a maximum when *w*_2_ = 288 nm and *h*_2_ = 120 nm. For such optimal parameters, extinction ratio *E*_*R*_ also reaches a maximum (2144), and intensities of rightward SPPs *I*_right_ is close to its maximum(*I*_right_/*I*_right,max_ = 0.8), simultaneously. We note that *I*_right_ reaches a maximum at *w*_2_ = 310 nm, *h*_2_ = 100 nm, while *E*_*R*_ is not high enough (*E*_*R*_ = 13). Hence, in the followings, we focus on structures with *w*_2_ = 288 nm and *h*_2_ = 120 nm.

**Figure 2:**
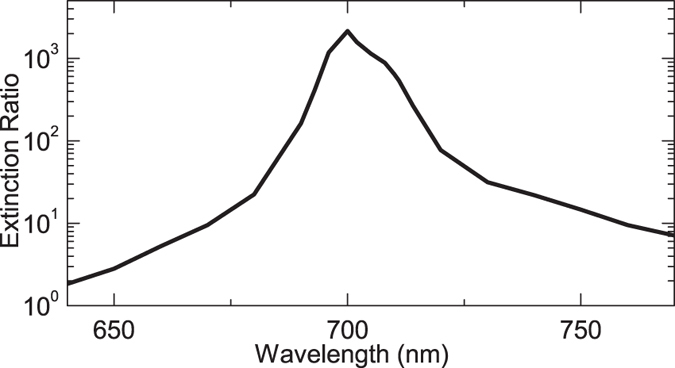
The extinction ratio *E*_*R*_ as a function of wavelength for the structure in Fig. 1(f).

Figure 1(f) shows the simulated profile of |**E**_*z*_|^2^/*p*^2^ for the above optimal structure. SPPs propagating to the right can be clearly observed, while leftward SPPs can hardly be seen. Such unidirectional launching of SPPs occurs in a broad frequency range. The extinction ratio *E*_*R*_ > 10 for wavelengths from 670 nm to 759 nm, and *E*_*R*_ reaches a maximum (2144) at *λ* = 700 nm [Fig. 2]. It is interesting to note that, if the slit is replaced by a groove (so that a groove-groove structure is formed) and illuminated from the top^22^,^23^, both rightward and leftward unidirectional SPPs can occur at different wavelengths. But in our slit-groove structure, only rightward unidirectional SPPs can occur^24^.

### A plasmonic lens with directional excitation of SPPs and its performance at different incident polarizations

**Figure 3:**
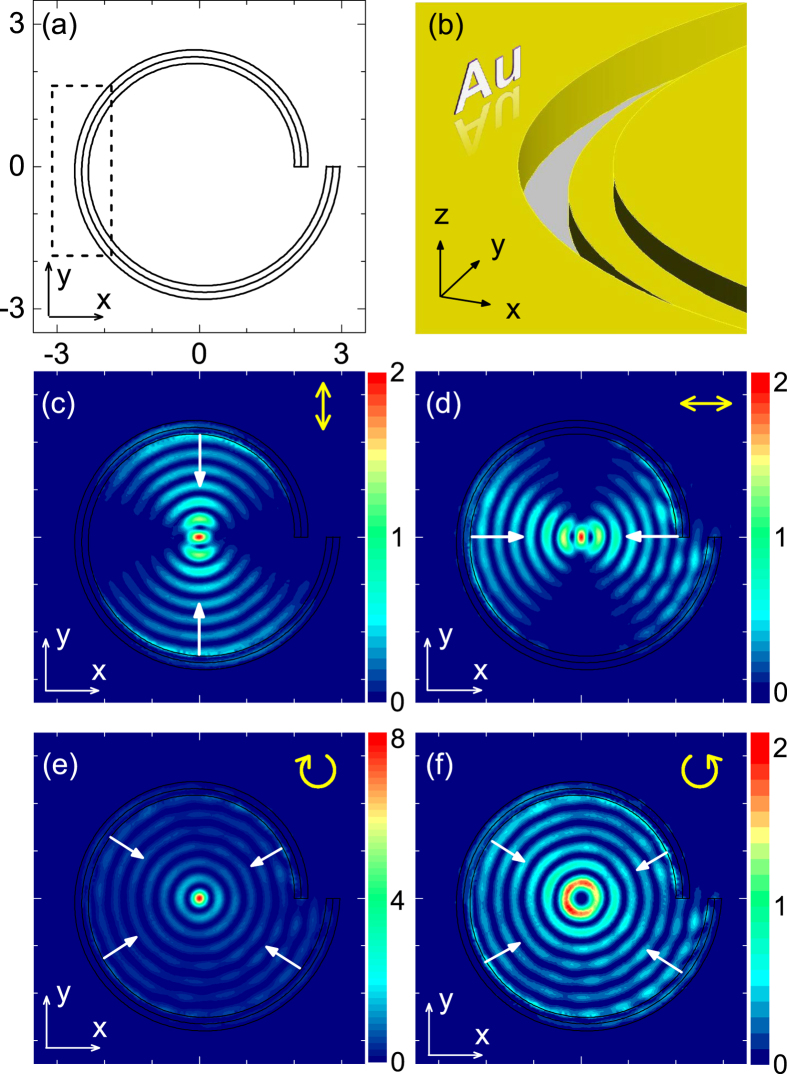
A plasmonic lens based on directional SPP excitation. (**a**) Top view of a plat Au film with a slit that has the same cross-sectional configuration as that in Fig. 1(b), but is curved into a right-handed Archimedes’ spiral shape in the *x*-*y* plane. The slit is illuminated from the back side by a Gaussian light beam with wavelength *λ* = 700 nm, width of *w*_*g*_ = 7 *μ*m, and |**E**| = *p* in center. (**b**) 3D plot of the area that is outlined by the dashed box in (**a**). (**c**) Simulated profile of |**E**_*z*_|^2^/*p*^2^ at a plane 50 nm above the Au film in (**a**). Here, a 7 *μ*m × 7 *μ*m area is shown and the incident light has *y*-polarization. (**d–f**) The same as (**c**) but for incident light with *x*-, left-handed, and right-handed polarizations, respectively.

The above asymmetric Au slit is along the *y* direction, which can be used to generate SPPs propagate along +*x* direction. If the slit is curled into a right-handed Archimedes’ spiral shape in the *x*-*y* plane and the auxiliary groove is located at the inside of the spiral [Fig. 3(a,b)], SPPs can be further focused into the central area of the spiral. The inner boundary of the slit is given by:


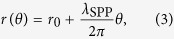


where *r*_0_ = 2 *μ*m, and *r* and *θ* are cylindrical coordinates. When Eq. (3) is satisfied, the antipodal points (*i.e*. points with *θ* = *θ*_1_ and *θ* = *θ*_1_ + *π*) at the spiral slit can generate inphase SPPs at the center of the spiral (*r* = 0) when the incident light has a linear polarization. We note that, when the slit is curled into a circle, this constructive interference cannot be achieved unless radially (rather than linear) polarized light is impinged on the structure^23^,^26^,^27^. Since such a radially polarized light beam should be centered at the center of the lens^26^,^27^, it cannot work in the lens array studied below.

We do simulations for the above plasmonic lens, which is illuminated normally from the back side by a Gaussian light beam. The incident light beam has a wavelength *λ* = 700 nm, width of *w*_*g*_ = 7 *μ*m, |**E**| = *p*, and center at the origin (*r* = 0). Figure 3(c) shows the simulated profile of |**E**_*z*_|^2^/*p*^2^ at a plane 50 nm above the Au film for the incidence with *y*-polarization. We can see that SPPs are excited at certain parts of the lens (*π*/4 < *θ* < 3*π*/4 or 5*π*/4 < *θ* < 7*π*/4, approximately). For the other parts, the angle is small between the slit and the *E*-field of the incident light, so that SPPs can hardly be launched. Besides, due to the introduction of auxiliary grooves, only inward SPPs are excited, which propagate to the center of the lens and form a hot spot. Similar phenomena can also be observed for incidence with *x*-polarization [Fig. 3(d)].

We have also studied the incidence with circular polarizations [Fig. 3(e,f)]. Light with left-handed polarization can be decomposed into *x*- and *y*-polarized light (**E**_left_ = *E*(**e**_*x*_ + *i***e**_**y**_)), which can generate SPPs with inphase **E**_**z**_ at the center of the lens. Such constructive interference forms a hot spot at the center of the lens [Fig. 3(f)]. Similar analyses have also be done for the right-handed incident light with **E**_right_ = *E*(**e**_*x*_ − *i***e**_**y**_). A dark spot appears at the center of the lens [Fig. 3(f)] due to the destructive interference. But a bright ring occurs around the dark spot. The spiral plasmonic lens exhibits different characteristics for left- and right-handed polarized incidence, and thus can serve as a device for discerning the handedness of circular polarization^4^,^5^,^28^.

### Comparison with imperfect plasmonic lenses

**Figure 4:**
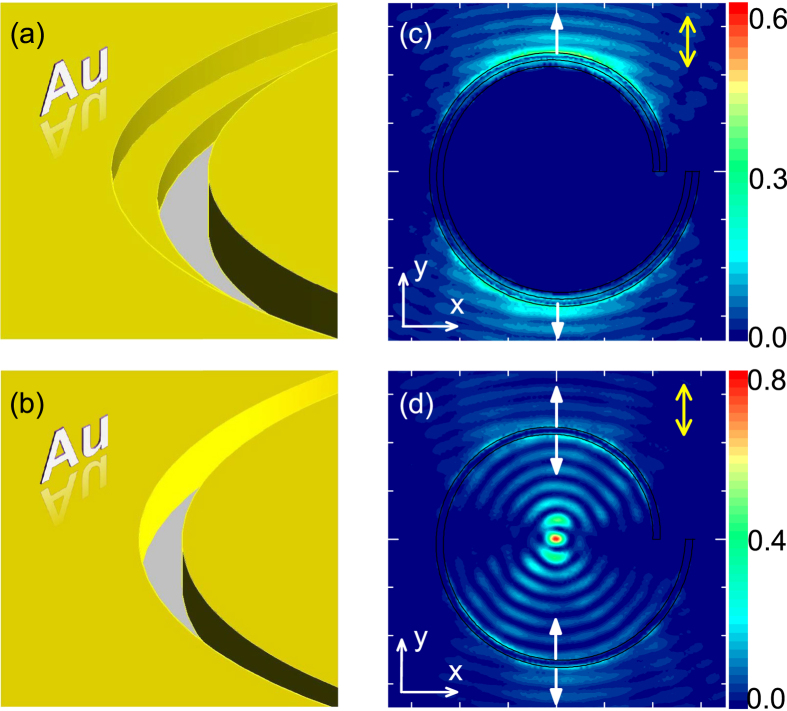
Imperfect plasmonic lenses. (**a**) The same as Fig. 3(b) but for a structure with the auxiliary groove at the outside of the spiral. (**b**) The same as Fig. 3(b) but for a structure without the auxiliary groove. (**c,d**) The same as Fig. 3(c) but for the structures in (**a,b**), respectively.

As comparison, we have also studied lenses with other structures as shown in Fig. 4. We can see that if the auxiliary groove is at the outside of the spiral [Fig. 4(a)], SPPs can propagate only outward while inward SPPs can hardly been observed. As a result, no hot spot is observed at the center of the lens [Fig. 4(c)]. When the auxiliary groove is not introduced [Fig. 4(b)], inward and outward SPPs can be excited simultaneously, forming a hot spot at the center of the lens [Fig. 4(d)]. However, due to the existence of outward SPPs, the intensity of the hot spot is only 1/2.5 times of that in Fig. 3(c). The same difference (1/2.5 times) also occur for circularly polarized illuminations. We note that for the inner side of the spiral, the thickness of the Au film decreases from *h*_1_ to *h*_1_ − *h*_2_ when comparing the structure in Fig. 3(b) to that in Fig. 4(b). This can also influence the intensity of the hot spot in Fig. 3(c), so that the observed enhancement (2.5 times) deviates from 2 times.

### Plasmonic lens array

**Figure 5:**
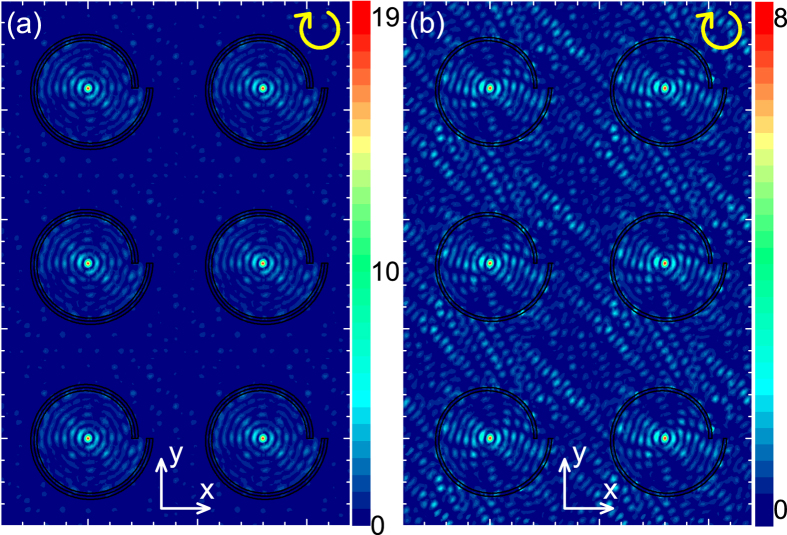
Performance of two plasmonic lens arrays. (**a**) The same as Fig. 3(e) but for a periodic array of spirals illuminated by a plane wave from the back side. The periods of array are 8 *μ*m in both the *x* and *y* directions. The intensity is shown in a 16 *μ*m × 24 *μ*m area. (**b**) The same as (**a**) but for structures without auxiliary grooves.

A plasmonic lens array may find applications in maskless lithography and multiple pixel^9^. Figure 5 demonstrates the performance for arrays of our and conventional plasmonic lenses. Here, an 8 *μ*m × 8 *μ*m area is simulated with using periodic boundary conditions and plane-wave incidence. For the conventional lens array, the interference of SPPs is obvious in the area outside lenses [Fig. 5(b)]. Such interference, which may be disadvantageous to applications, are not found in our plasmonic lens array [Fig. 5(a)]. Because of the elimination of field outside lenses, the field intensity at the center of our lens is much stronger than that of conventional lens. Thus, our lens array could have improved performance in the above-mentioned applications.

## Discussion

We have presented a spiral plasmonic lens based on a metallic slit with an auxiliary groove. When the lens is illumined normally from the back side, no outward SPPs can be excited, while only inward SPPs can be launched and then focused into the center of the lens. This phenomenon occurs for all the circular and linear polarizations of incidence. When optimal groove parameters are applied, the intensity at the focal spot of our lens can be 2.5 times of that without an auxiliary groove for both linearly and circularly polarized illuminations.

## Additional Information

**How to cite this article**: Guo, Q. *et al*. A spiral plasmonic lens with directional excitation of surface plasmons. *Sci. Rep*. **6**, 32345; doi: 10.1038/srep32345 (2016).
